# Editorial: Go with the vet-flow! The current uses and new frontiers of flow cytometry in veterinary sciences

**DOI:** 10.3389/fvets.2024.1532735

**Published:** 2024-12-05

**Authors:** Fulvio Riondato, Alberto Alvarez-Barrientos, Francesco Grandoni

**Affiliations:** ^1^Department of Veterinary Sciences, School of Agriculture and Veterinary Medicine, University of Turin, Grugliasco, Italy; ^2^Bioscience Applied Techniques Facility (STAB), Universidad de Extremadura, Badajoz, Spain; ^3^Research Centre for Animal Production and Aquaculture, Consiglio per la Ricerca in Agricoltura e l'Analisi dell'Economia Agraria (CREA), Monterotondo, Italy

**Keywords:** flow cytometry, veterinary, dog, cat, marine mammals, camel, chicken, pig

Flow cytometry has captured the interest of veterinary scientists since the late 1970s. Despite advancements, the gap between veterinary and biomedical flow cytometry remains significant. The present Research Topic was primarily developed to address two questions. The first question, posed by the scientist, is: “*Veterinary sciences and flow cytometry—what are the connections between these two fields?”* A search for “flow cytometry” (in the title, abstract, or keywords) within the Scopus database yields 6,797 publications in journals classified under the subject area “Veterinary” (at November 19, 2024). These publications are predominantly co-classified in medical and biological subject areas (“Immunology and Microbiology”, “Agricultural and Biological Sciences”, “Medicine”, “Biochemistry, Genetics and Molecular Biology”), although other fields are also represented (“Pharmacology, Toxicology and Pharmaceutics”, “Environmental Science”, “Health Professions”, “Nursing”, and “Social Sciences”). The earliest paper included in this search was published in 1980 (1). Although some earlier studies exist (2, 3), they are published in journals not classified within the “Veterinary” subject area. Since then, the number of publications has shown steady growth, reaching 469 in 2024, with a significant increase starting in 2020. Against this background, this Research Topic aims to “take the pulse” of flow cytometry in the veterinary field today and to present findings from recent studies in relevant areas.

The second question, posed by the cytometrist, is: “*Where should I publish the results of my work?”* The search in the Scopus database reveals that the 6,797 papers have been published in over 150 different journals. This diversity reflects the lack of journals or journal sections specifically dedicated to flow cytometry within the veterinary sciences. Nonetheless, a minority of papers have been published in journals specifically focused on flow cytometry. Specifically, a Scopus search for the word “cytometry” in the journal title, combined with “Veter^*^” or an animal species in the “Article Title, Abstract, Keywords” field, resulted in over 150 papers in 6 different journals, most of which are published in *Cytometry*. However, none of these journals are classified under the “Veterinary” subject area. As a result, authors are compelled to publish in journals focused on specific disciplines or, if they choose to publish in flow cytometry journals, they must forego making a direct contribution to the “official” veterinary field. This situation limits the development of a “vet-flow community” and hinders the growth of interest, expertise, collaboration, and thus, the achievement of meaningful results.

The Research Topic was open to studies from various disciplines to address these two aims. The 11 included articles provide a broad, albeit partial, overview depicting a highly diverse and dynamic field. They demonstrate that flow cytometry is currently used for studies on various animal species, applied to different biological samples, and serving multiple purposes. Studies encompass a range of species, including dogs, cats, pigs, dromedary camels, chickens, and marine mammals, with flow cytometric analyses performed on neoplastic masses, lymph nodes, peripheral blood, and cavity effusions, reporting results of cross-cutting interest. Sini et al. demonstrate that flow cytometry is a reliable alternative to immunohistochemistry for detecting cytokeratin, vimentin, and desmin in canine effusions, paving the way for including these markers in flow cytometric panels as a diagnostic tool to differentiate mesothelial, epithelial, and mesenchymal cells. The article by Ubiali et al. highlights the role of PD-L1 in the pathogenesis of canine lymphomas, revealing differences among B-cell, aggressive T-cell, and T-zone lymphomas. The study examines the surface membrane protein expression and relative mRNA levels in neoplastic cells, and the soluble protein concentration in plasma. Stokol et al. describe CD80 expression in leukocytes from canine peripheral blood and bone marrow, recommending its inclusion in flow cytometric immunophenotyping panels as a lineage marker for diagnosing acute myeloid leukemia. A method for evaluating Ki67 expression by flow cytometry in canine mast cell tumors is presented by Wu et al., offering oncologists a potential tool for prognostication. Rütgen et al. provide a significant case series of gastrointestinal lymphomas in cats, correlating multicolor flow cytometric analysis results with histopathologic classification and PCR clonality testing (PARR), and identifying unique immunophenotypes within B-cell and T-cell gastrointestinal neoplasms. An interesting study by Zwicklbauer et al. introduces a method for stabilizing whole blood from cats to enable long-term flow cytometric analyses, proving the reliability of quantifying T-helper cells, cytotoxic T-cells, B-cells, monocytes, and neutrophils up to 2 years post-sampling. Hussen et al. describe the application of a flow cytometric panel to analyze immune cell composition in the lymph node population compared to peripheral blood in dromedary camels, highlighting differences in lymphoid subset prevalence and specific marker expression. A multicolor approach for characterizing T CD4^+^ and CD8^+^ subsets in pigs aimed at studying activation-induced markers is reported by Moorton et al.. Härtle et al. provide an extensive overview of flow cytometry's potential to characterize different leukocyte populations in chickens, summarizing available reagents and guidelines for multicolor approaches. Finally, Felipo-Benavent, Mart-Romero et al. and Felipo-Benavent, Valls et al. focus on marine mammals in two separate articles, detailing flow cytometric methods for assessing phagocytic capacity and platelet function. They provide the first physiological values for various species, offering new tools for evaluating marine mammal health with potential clinical application in aquariums and other settings.

In addition to providing a snapshot of current flow cytometry applications in veterinary medicine and presenting recent findings across disciplines, this Research Topic could represent an important agora for all veterinary cytometrists regardless of their specific field. Considering the number and scientific quality of the manuscripts selected, we believe that the first issue has successfully achieved its objectives and laid the groundwork for a second volume. We hope this work will encourage interest and attract resources to support the continued growth of the “vet-flow” field in the future and help establish flow cytometry as an independent discipline within veterinary sciences too.
